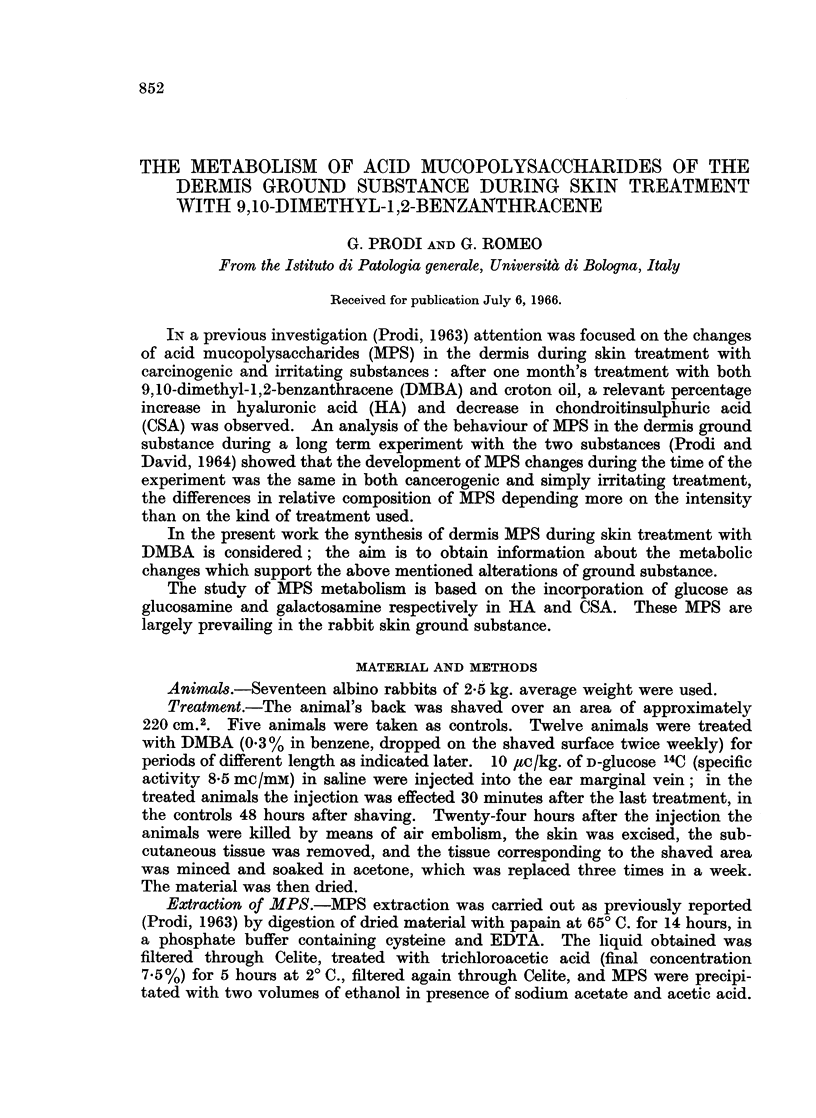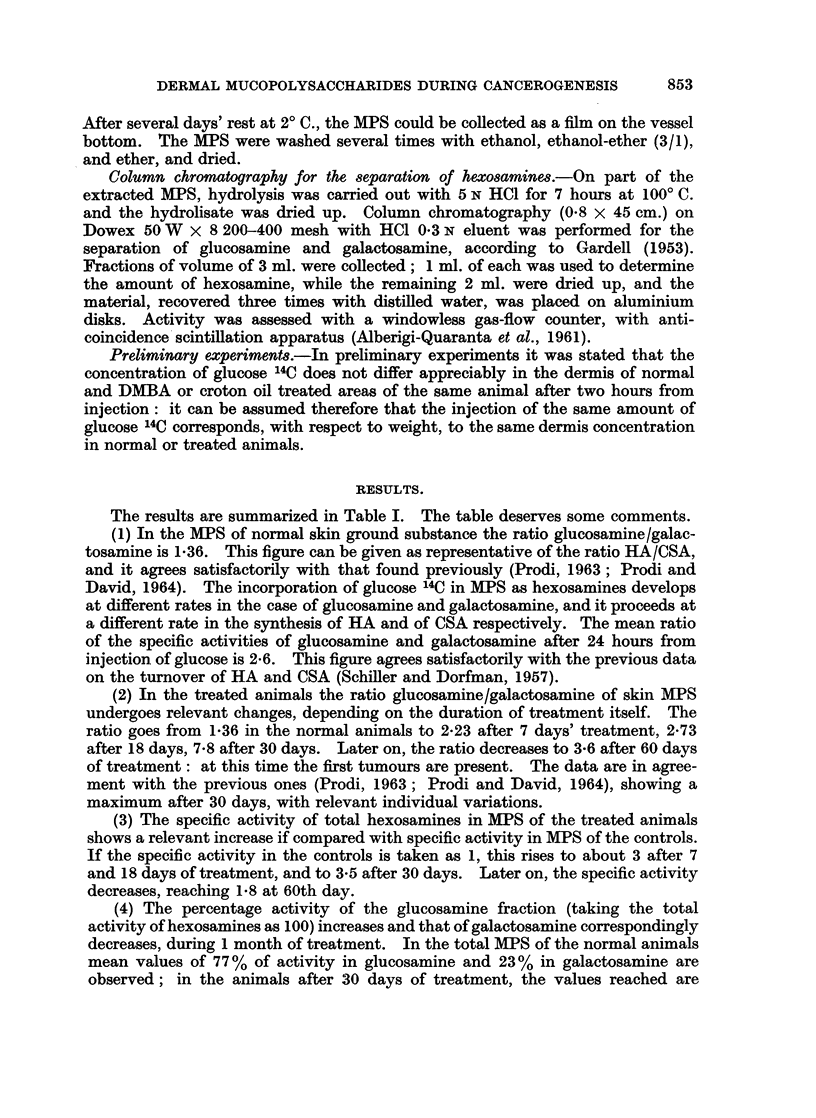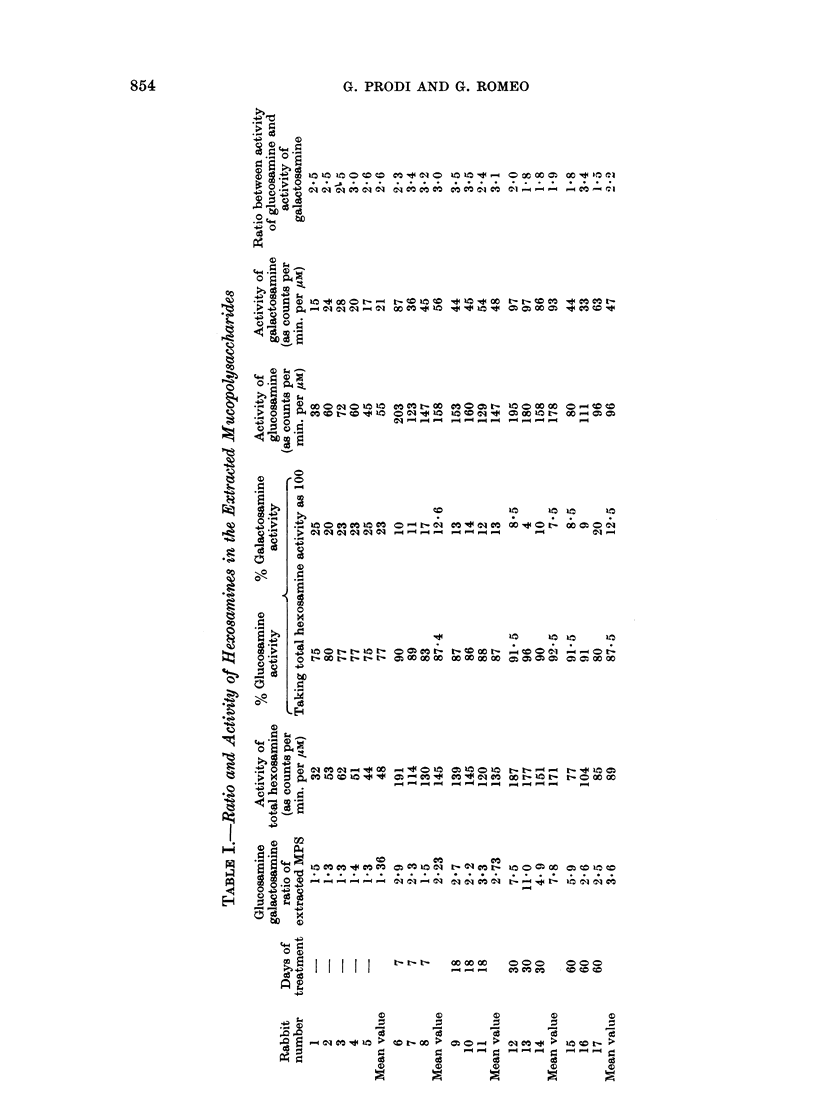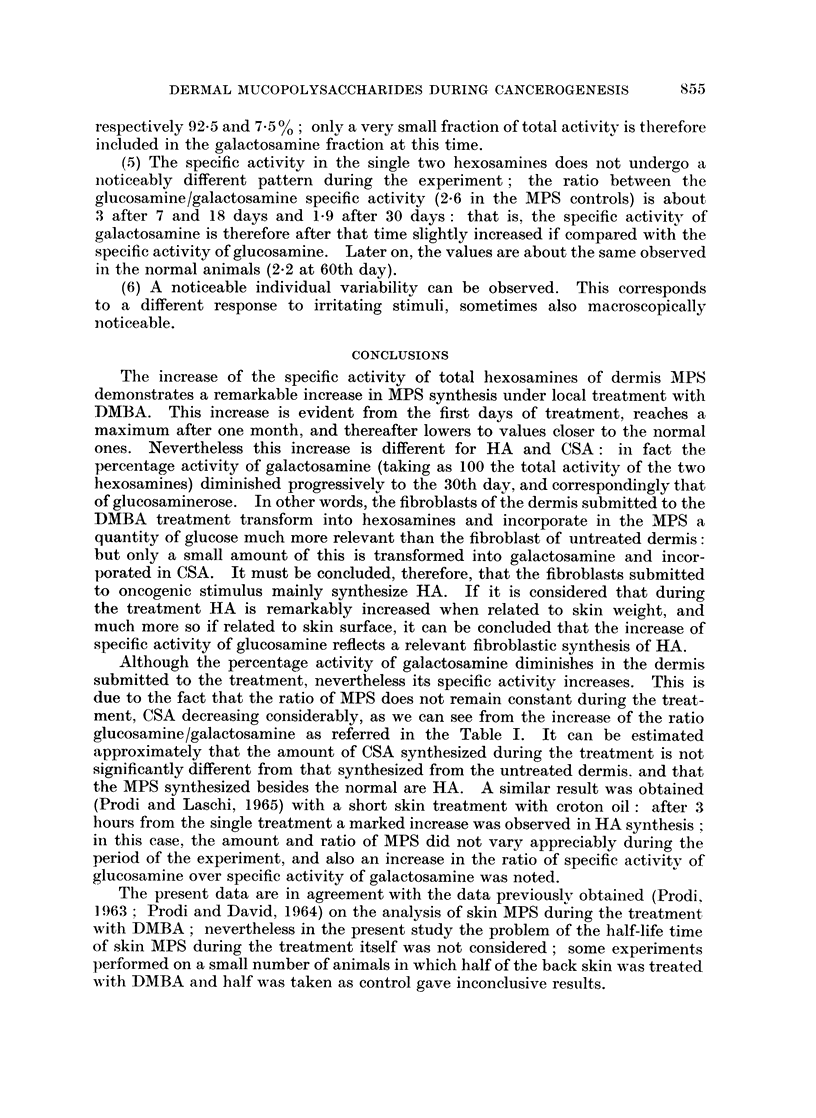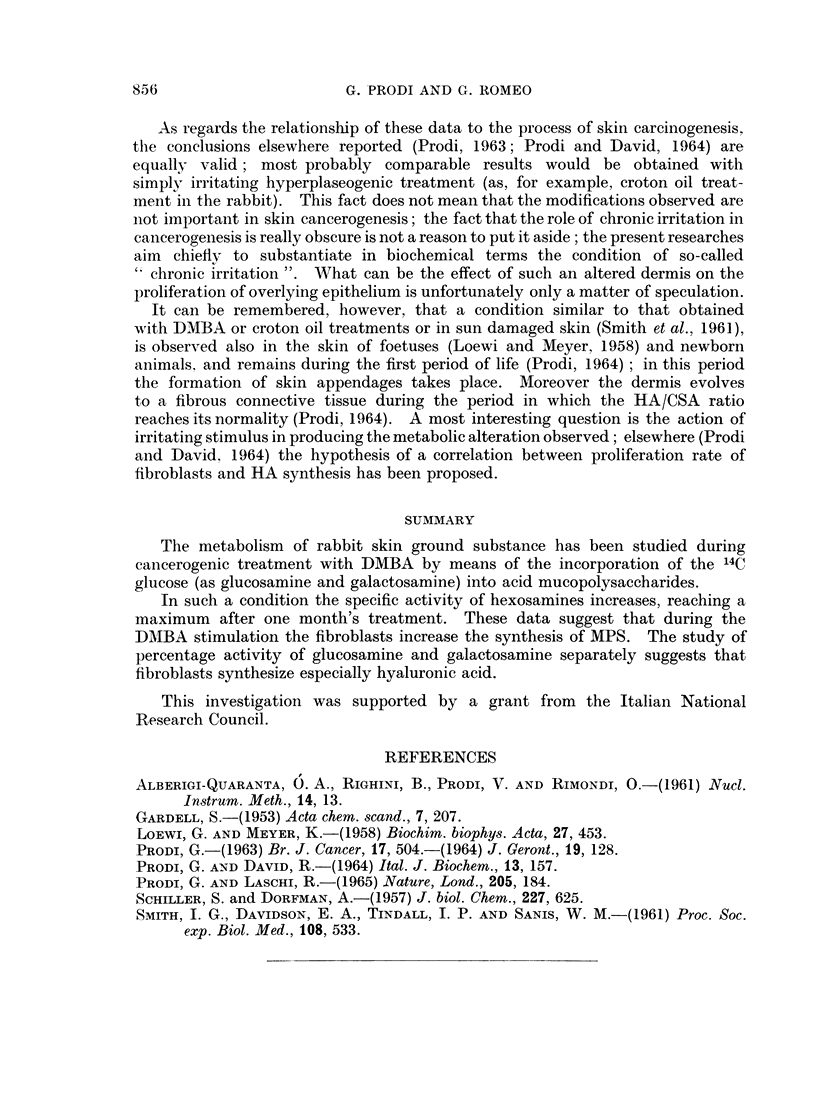# The metabolism of acid mucopolysaccharides of the dermis ground substance during skin treatment with 9,10-dimethyl-1,2-benzanthracene.

**DOI:** 10.1038/bjc.1966.98

**Published:** 1966-12

**Authors:** G. Prodi, G. Romeo


### THE METABOLISM OF ACID MUCOPOLYSACCHARIDES OF THE DERMIS GROUND SUBSTANCE DURING SKIN TREATMENT WITH 9,10-DIMETHYL-1,2-BENZANTHRACENE

G. PRODI AND G. ROMEO

*From the Istituto di Patologia generale, Università di Bologna, Italy*

Received for publication July 6, 1966.

IN a previous investigation (Prodi, 1963) attention was focused on the changes of acid mucopolysaccharides (MPS) in the dermis during skin treatment with carcinogenic and irritating substances: after one month's treatment with both 9,10-dimethyl-1,2-benzanthracene (DMBA) and croton oil, a relevant percentage increase in hyaluronic acid (HA) and decrease in chondroitinsulphuric acid (CSA) was observed. An analysis of the behaviour of MPS in the dermis ground substance during a long term experiment with the two substances (Prodi and David, 1964) showed that the development of MPS changes during the time of the experiment was the same in both cancerogenic and simply irritating treatment, the differences in relative composition of MPS depending more on the intensity than on the kind of treatment used.

In the present work the synthesis of dermis MPS during skin treatment with DMBA is considered; the aim is to obtain information about the metabolic changes which support the above mentioned alterations of ground substance.

The study of MPS metabolism is based on the incorporation of glucose as glucosamine and galactosamine respectively in HA and CSA. These MPS are largely prevailing in the rabbit skin ground substance.

### MATERIAL AND METHODS

*Animals.*—Seventeen albino rabbits of 2·5 kg. average weight were used.

*Treatment.*—The animal's back was shaved over an area of approximately 220 cm.$^2$. Five animals were taken as controls. Twelve animals were treated with DMBA (0·3% in benzene, dropped on the shaved surface twice weekly) for periods of different length as indicated later. 10 $\mu$c/kg. of D-glucose $^{14}$C (specific activity 8·5 mc/mM) in saline were injected into the ear marginal vein; in the treated animals the injection was effected 30 minutes after the last treatment, in the controls 48 hours after shaving. Twenty-four hours after the injection the animals were killed by means of air embolism, the skin was excised, the subcutaneous tissue was removed, and the tissue corresponding to the shaved area was minced and soaked in acetone, which was replaced three times in a week. The material was then dried.

*Extraction of MPS.*—MPS extraction was carried out as previously reported (Prodi, 1963) by digestion of dried material with papain at 65° C. for 14 hours, in a phosphate buffer containing cysteine and EDTA. The liquid obtained was filtered through Celite, treated with trichloroacetic acid (final concentration 7·5%) for 5 hours at 2° C., filtered again through Celite, and MPS were precipitated with two volumes of ethanol in presence of sodium acetate and acetic acid.

After several days' rest at 2° C., the MPS could be collected as a film on the vessel bottom. The MPS were washed several times with ethanol, ethanol-ether (3/1), and ether, and dried.

*Column chromatography for the separation of hexosamines.*—On part of the extracted MPS, hydrolysis was carried out with 5 N HCl for 7 hours at 100° C. and the hydrolisate was dried up. Column chromatography (0·8 × 45 cm.) on Dowex 50 W × 8 200–400 mesh with HCl 0·3 N eluent was performed for the separation of glucosamine and galactosamine, according to Gardell (1953). Fractions of volume of 3 ml. were collected ; 1 ml. of each was used to determine the amount of hexosamine, while the remaining 2 ml. were dried up, and the material, recovered three times with distilled water, was placed on aluminium disks. Activity was assessed with a windowless gas-flow counter, with anti-coincidence scintillation apparatus (Alberigi-Quaranta *et al.*, 1961).

*Preliminary experiments.*—In preliminary experiments it was stated that the concentration of glucose $^{14}C$ does not differ appreciably in the dermis of normal and DMBA or croton oil treated areas of the same animal after two hours from injection : it can be assumed therefore that the injection of the same amount of glucose $^{14}C$ corresponds, with respect to weight, to the same dermis concentration in normal or treated animals.

### RESULTS.

The results are summarized in Table I. The table deserves some comments.

(1) In the MPS of normal skin ground substance the ratio glucosamine/galactosamine is 1·36. This figure can be given as representative of the ratio HA/CSA, and it agrees satisfactorily with that found previously (Prodi, 1963 ; Prodi and David, 1964). The incorporation of glucose $^{14}C$ in MPS as hexosamines develops at different rates in the case of glucosamine and galactosamine, and it proceeds at a different rate in the synthesis of HA and of CSA respectively. The mean ratio of the specific activities of glucosamine and galactosamine after 24 hours from injection of glucose is 2·6. This figure agrees satisfactorily with the previous data on the turnover of HA and CSA (Schiller and Dorfman, 1957).

(2) In the treated animals the ratio glucosamine/galactosamine of skin MPS undergoes relevant changes, depending on the duration of treatment itself. The ratio goes from 1·36 in the normal animals to 2·23 after 7 days' treatment, 2·73 after 18 days, 7·8 after 30 days. Later on, the ratio decreases to 3·6 after 60 days of treatment : at this time the first tumours are present. The data are in agreement with the previous ones (Prodi, 1963 ; Prodi and David, 1964), showing a maximum after 30 days, with relevant individual variations.

(3) The specific activity of total hexosamines in MPS of the treated animals shows a relevant increase if compared with specific activity in MPS of the controls. If the specific activity in the controls is taken as 1, this rises to about 3 after 7 and 18 days of treatment, and to 3·5 after 30 days. Later on, the specific activity decreases, reaching 1·8 at 60th day.

(4) The percentage activity of the glucosamine fraction (taking the total activity of hexosamines as 100) increases and that of galactosamine correspondingly decreases, during 1 month of treatment. In the total MPS of the normal animals mean values of 77% of activity in glucosamine and 23% in galactosamine are observed ; in the animals after 30 days of treatment, the values reached are

TABLE I.—*Ratio and Activity of Hexosamines in the Extracted Mucopolysaccharides*

| Rabbit number | Days of treatment | Glucosamine galactosamine ratio of extracted MPS | Activity of total hexosamine (as counts per min. per μM) | % Glucosamine activity (Taking total hexosamine activity as 100) | % Galactosamine activity (Taking total hexosamine activity as 100) | Activity of glucosamine (as counts per min. per μM) | Activity of galactosamine (as counts per min. per μM) | Ratio between activity of glucosamine and activity of galactosamine |
|---|---|---|---|---|---|---|---|---|
| 1 | — | 1·5 | 32 | 75 | 25 | 38 | 15 | 2·5 |
| 2 | — | 1·3 | 53 | 80 | 20 | 60 | 24 | 2·5 |
| 3 | — | 1·3 | 62 | 77 | 23 | 72 | 28 | 2·5 |
| 4 | — | 1·4 | 51 | 77 | 23 | 60 | 20 | 3·0 |
| 5 | — | 1·3 | 44 | 75 | 25 | 45 | 17 | 2·6 |
| Mean value | | 1·36 | 48 | 77 | 23 | 55 | 21 | 2·6 |
| 6 | 7 | 2·9 | 191 | 90 | 10 | 203 | 87 | 2·3 |
| 7 | 7 | 2·3 | 114 | 89 | 11 | 123 | 36 | 3·4 |
| 8 | 7 | 1·5 | 130 | 83 | 17 | 147 | 45 | 3·2 |
| Mean value | | 2·23 | 145 | 87·4 | 12·6 | 158 | 56 | 3·0 |
| 9 | 18 | 2·7 | 139 | 87 | 13 | 153 | 44 | 3·5 |
| 10 | 18 | 2·2 | 145 | 86 | 14 | 160 | 45 | 3·5 |
| 11 | 18 | 3·3 | 120 | 88 | 12 | 129 | 54 | 2·4 |
| Mean value | | 2·73 | 135 | 87 | 13 | 147 | 48 | 3·1 |
| 12 | 30 | 7·5 | 187 | 91·5 | 8·5 | 195 | 97 | 2·0 |
| 13 | 30 | 11·0 | 177 | 96 | 4 | 180 | 97 | 1·8 |
| 14 | 30 | 4·9 | 151 | 90 | 10 | 158 | 86 | 1·8 |
| Mean value | | 7·8 | 171 | 92·5 | 7·5 | 178 | 93 | 1·9 |
| 15 | 60 | 5·9 | 77 | 91·5 | 8·5 | 80 | 44 | 1·8 |
| 16 | 60 | 2·6 | 104 | 91 | 9 | 111 | 33 | 3·4 |
| 17 | 60 | 2·5 | 85 | 80 | 20 | 96 | 63 | 1·5 |
| Mean value | | 3·6 | 89 | 87·5 | 12·5 | 96 | 47 | 2·2 |

respectively 92·5 and 7·5% ; only a very small fraction of total activity is therefore included in the galactosamine fraction at this time.

(5) The specific activity in the single two hexosamines does not undergo a noticeably different pattern during the experiment ; the ratio between the glucosamine/galactosamine specific activity (2·6 in the MPS controls) is about 3 after 7 and 18 days and 1·9 after 30 days : that is, the specific activity of galactosamine is therefore after that time slightly increased if compared with the specific activity of glucosamine. Later on, the values are about the same observed in the normal animals (2·2 at 60th day).

(6) A noticeable individual variability can be observed. This corresponds to a different response to irritating stimuli, sometimes also macroscopically noticeable.

### CONCLUSIONS

The increase of the specific activity of total hexosamines of dermis MPS demonstrates a remarkable increase in MPS synthesis under local treatment with DMBA. This increase is evident from the first days of treatment, reaches a maximum after one month, and thereafter lowers to values closer to the normal ones. Nevertheless this increase is different for HA and CSA : in fact the percentage activity of galactosamine (taking as 100 the total activity of the two hexosamines) diminished progressively to the 30th day, and correspondingly that of glucosaminerose. In other words, the fibroblasts of the dermis submitted to the DMBA treatment transform into hexosamines and incorporate in the MPS a quantity of glucose much more relevant than the fibroblast of untreated dermis : but only a small amount of this is transformed into galactosamine and incorporated in CSA. It must be concluded, therefore, that the fibroblasts submitted to oncogenic stimulus mainly synthesize HA. If it is considered that during the treatment HA is remarkably increased when related to skin weight, and much more so if related to skin surface, it can be concluded that the increase of specific activity of glucosamine reflects a relevant fibroblastic synthesis of HA.

Although the percentage activity of galactosamine diminishes in the dermis submitted to the treatment, nevertheless its specific activity increases. This is due to the fact that the ratio of MPS does not remain constant during the treatment, CSA decreasing considerably, as we can see from the increase of the ratio glucosamine/galactosamine as referred in the Table I. It can be estimated approximately that the amount of CSA synthesized during the treatment is not significantly different from that synthesized from the untreated dermis. and that the MPS synthesized besides the normal are HA. A similar result was obtained (Prodi and Laschi, 1965) with a short skin treatment with croton oil : after 3 hours from the single treatment a marked increase was observed in HA synthesis ; in this case, the amount and ratio of MPS did not vary appreciably during the period of the experiment, and also an increase in the ratio of specific activity of glucosamine over specific activity of galactosamine was noted.

The present data are in agreement with the data previously obtained (Prodi, 1963 ; Prodi and David, 1964) on the analysis of skin MPS during the treatment with DMBA ; nevertheless in the present study the problem of the half-life time of skin MPS during the treatment itself was not considered ; some experiments performed on a small number of animals in which half of the back skin was treated with DMBA and half was taken as control gave inconclusive results.

As regards the relationship of these data to the process of skin carcinogenesis, the conclusions elsewhere reported (Prodi, 1963; Prodi and David, 1964) are equally valid; most probably comparable results would be obtained with simply irritating hyperplaseogenic treatment (as, for example, croton oil treatment in the rabbit). This fact does not mean that the modifications observed are not important in skin cancerogenesis; the fact that the role of chronic irritation in cancerogenesis is really obscure is not a reason to put it aside; the present researches aim chiefly to substantiate in biochemical terms the condition of so-called " chronic irritation ". What can be the effect of such an altered dermis on the proliferation of overlying epithelium is unfortunately only a matter of speculation. It can be remembered, however, that a condition similar to that obtained with DMBA or croton oil treatments or in sun damaged skin (Smith *et al.*, 1961), is observed also in the skin of foetuses (Loewi and Meyer, 1958) and newborn animals, and remains during the first period of life (Prodi, 1964); in this period the formation of skin appendages takes place. Moreover the dermis evolves to a fibrous connective tissue during the period in which the HA/CSA ratio reaches its normality (Prodi, 1964). A most interesting question is the action of irritating stimulus in producing the metabolic alteration observed; elsewhere (Prodi and David, 1964) the hypothesis of a correlation between proliferation rate of fibroblasts and HA synthesis has been proposed.

### SUMMARY

The metabolism of rabbit skin ground substance has been studied during cancerogenic treatment with DMBA by means of the incorporation of the $^{14}C$ glucose (as glucosamine and galactosamine) into acid mucopolysaccharides.

In such a condition the specific activity of hexosamines increases, reaching a maximum after one month's treatment. These data suggest that during the DMBA stimulation the fibroblasts increase the synthesis of MPS. The study of percentage activity of glucosamine and galactosamine separately suggests that fibroblasts synthesize especially hyaluronic acid.

This investigation was supported by a grant from the Italian National Research Council.

### REFERENCES

ALBERIGI-QUARANTA, Ó. A., RIGHINI, B., PRODI, V. AND RIMONDI, O.—(1961) *Nucl. Instrum. Meth.*, **14**, 13.

GARDELL, S.—(1953) *Acta chem. scand.*, **7**, 207.

LOEWI, G. AND MEYER, K.—(1958) *Biochim. biophys. Acta*, **27**, 453.

PRODI, G.—(1963) *Br. J. Cancer*, **17**, 504.—(1964) *J. Geront.*, **19**, 128.

PRODI, G. AND DAVID, R.—(1964) *Ital. J. Biochem.*, **13**, 157.

PRODI, G. AND LASCHI, R.—(1965) *Nature, Lond.*, **205**, 184.

SCHILLER, S. and DORFMAN, A.—(1957) *J. biol. Chem.*, **227**, 625.

SMITH, I. G., DAVIDSON, E. A., TINDALL, I. P. AND SANIS, W. M.—(1961) *Proc. Soc. exp. Biol. Med.*, **108**, 533.